# 

**Published:** 1886-04

**Authors:** 


### CONTENTS:

Article I—New Experiments and Observations on Hydrobromic Ether or the Bromide of Ethyl as an Anaesthetic. By Laurence Turnbull, M. D.....529

Art. II—Caries and Necrosis of Alveoli of Right Superior Maxilla and Chronic Inflammation of Antrum, due to Excessive Salivary Concretions and a Neglected Pulpless Molar By B. Merrill Hopkinson, D. D. S. M. D 548

Art. III—The Wisdom Teeth. By J. D. Thomas, D. D. S.....554

Art. IV—Effects of Amalgam Fillings upon the System. By E A Bogue, M. D....563

Art. V—The Law Regulating the Practice of Dentistry in the State of Virginia.....566

Art. VI—German Trial for Holding an Irregular Diploma. By F. Zeitung.........568

Chicago College of Dental Surgery.....569

To State and Local Dental Societies.....570

To the Members of the American Dental Association.....571

### EDITORIAL, ETC.

The Maryland Dental Law Amendments.....571

The Coming Meeting of the American Dental Association.....572

Dr B. H. Teague's Depressed Engine Disks.....572

### MONTHLY SUMMARY.

Prevention of Bad Teeth.....573

Prosecution of a Dentist.....574

The Fluctuations in Cocaine.....575

Mr. Jonathan Hutchinson.....576

